# Reply to Franzini et al. The Translational Medicine Regarding Ozone in Saline Solutions. Comment on “Armeli et al. Ozone Saline Solution Polarizes Microglial Cells Towards an Anti-Inflammatory Phenotype. *Molecules* 2025, *30*, 3932”

**DOI:** 10.3390/molecules31111825

**Published:** 2026-05-26

**Authors:** Federica Armeli, Beatrice Mengoni, Martina Menin, Gregorio Martínez-Sánchez, Mauro Martinelli, Maurizio Maggiorotti, Rita Businaro

**Affiliations:** 1 Department of Medico-Surgical Sciences and Biotechnologies, Sapienza University of Rome, 04100 Latina, Italy; federica.armeli@uniroma1.it (F.A.); beatrice.mengoni@uniroma1.it (B.M.); menin_martina@yahoo.it (M.M.); 2 Independent Researcher, 60126 Ancona, Italy; gregorcuba@yahoo.it; 3 Ozone Therapy Unit, Department of Biomedical Sciences, Ospedale San Pietro Fatebenefratelli, 00189 Rome, Italy; mauro.martinelli53@gmail.com; 4 Italian College of Oxygen-Ozone Therapy (CIO3), 00186 Rome, Italy; maggiorotti@gmail.com

**Keywords:** ozonized saline solution, microglial polarization, redox signaling, in vitro models, translational medicine

## Abstract

In this Reply, we address the criticisms raised by Franzini, Valdenassi, and Chirumbolo concerning our study on the effects of ozonized saline solution (O3SS) on microglial polarization and endothelial responses in vitro. We clarify that the primary aim of the original work was mechanistic, relying on rigorously controlled cellular models that are universally recognized as essential preclinical tools in translational medicine. We reaffirm the validity of our experimental approach, including the preparation and characterization of O3SS based on empirically validated methodologies, direct ozone quantification, and standardized protocols consistent with the existing literature and clinical practice. Concerns regarding ozone chemistry, dose relevance, and hypochlorite formation are addressed through analytical validation, biological threshold considerations, and the use of certified assays. We further justify the choice of BV2 microglia and HUVEC cells as established and widely used models for investigating inflammatory and vascular pathways under reproducible conditions. Statistical analyses, gene expression interpretation, and the absence of comparative pharmacological agents are discussed in the context of the study’s focused objectives. Finally, we place our findings within the established framework of ozone as an indirect pro-oxidant that elicits adaptive redox signaling (“oxidative eustress”), emphasizing the translational relevance of in vitro systems for elucidating early mechanistic events. Overall, we maintain that our study provides a robust, balanced, and evidence-based contribution to the understanding of ozone-derived redox biology.

## 1. Introduction

We would like to thank you for giving us the opportunity to respond to the criticisms raised by Dr. Franzini and coworkers to our paper entitled “Ozone Saline Solution Polarizes Microglial Cells Towards an Anti-Inflammatory Phenotype” published in *Molecules*, 2025; 30(19): 3932; doi: 10.3390/molecules30193932 [1]. We begin by saying that the main criticism concerns the entire approach of the study based on in vitro cellular models that allow analyses under strictly controlled and reproducible conditions. Franzini et al. deny the validity of the in vitro studies [2], which are preclinical and represent the sine qua non condition for further assessments on animal models as well as human subjects (translational medicine). We take this opportunity to outline some fundamental principles of good research practices and good clinical practices, as reported by various national and international organizations. Research integrity is crucial to preserving the trustworthiness of the research system and its results, as is good clinical practice, to ensure the protection of human subjects and the reliability of results (https://allea.org/wp-content/uploads/2023/06/European-Code-of-Conduct-Revised-Edition-2023.pdf accessed on 1 July 2025) [3].

In this connection, preclinical research is the experimental phase preceding human trials, essential for evaluating the safety, toxicity, and efficacy of new drugs or therapies. It uses in vitro (cell cultures) and in vivo (animal models) tests to study molecular and biological behavior to ensure safety and benefit for patients, which reduces risks prior to human clinical trials. It produces essential data, such as toxicity for pharmacokinetic (absorption, distribution, metabolism, and excretion) and pharmacodynamic studies. Preclinical research is primarily aimed at excluding the toxicity of new drugs/therapeutic procedures, aiming at avoiding useless, if not harmful, experiments directly on human subjects.

To clarify our position, however, we absolutely agree that human subjects/patients constitute complex systems, characterized by numerous variables, both physiological and pathological, and therefore, in vitro studies are necessarily preclinical in that they cannot replace in vivo studies. Nonetheless, they are systems that, if used rigorously, as was the case in our study, can help elucidate some pathways involved in homeostasis or, instead, in pathological imbalances. We note that the Comment raises substantive concerns about the study’s validity. While we respect the authors’ perspective, we also wish to recall that the original manuscript underwent peer review and was accepted for publication. Our aim here is to address the scientific issues raised, rather than to evaluate the appropriateness of the critics’ statements. Below, we respond point by point to all the criticisms raised against our study.

## 2. Preparation and Characterization of Ozonized Saline Solution

The preparation of the ozonized saline solution (O3SS) followed a previously validated methodology. Obviously, it was not possible to directly add ozone to a volume less than the milliliter contained in the wells of the microplate in which the cells are grown. The ozone was added to 500 mL of saline, the container of which was connected to the ozone dispenser, and then added to the cells. This statement appears to reflect a misunderstanding of our reported methods. The ozone was added to 500 mL of saline, not directly to the microplate wells. Therefore, the concern regarding small-volume vs. bulk-volume behavior does not apply to our experimental setup. We clarify this point to avoid further confusion.

Empirical Calibration of Ozone Concentrations: As clearly stated in the Materials and Methods Section, the preparation of the ozonized saline solution (O3SS) was based on a previously validated and referenced methodology. The ozone concentrations in the saline solution were measured experimentally using UV spectrophotometry—the gold standard for quantifying dissolved ozone—not assumed theoretically. The established correlation between the generator output and the final dissolved concentration (10% of the nominal value) was determined empirically and accounts for factors such as ozone decay, gas–liquid transfer efficiency, and equilibrium conditions. Therefore, the claim that the study relies on “untenable equivalence” overlooks this foundational calibration step. Controlled and Clinically Relevant Preparation: The O3SS was generated using a certified medical ozone generator (WeZONO) under controlled and reproducible conditions, adhering to ISCO3 quality criteria. The solution was prepared in macroscopic volumes (sufficient for all replicates) and then diluted into culture media, ensuring consistency across experimental wells. This method directly mimics the preparation of ozone saline for clinical infusion, where ozone is first dissolved in a larger saline volume before administration. Focus on Mechanistic Insight, Not Direct Extrapolation: The study was explicitly designed as a mechanistic investigation into the cellular and molecular responses to ozone-derived reactive species under defined in vitro conditions. Nowhere do we claim a one-to-one translation of in vitro doses to in vivo systemic effects. Instead, we use a controlled model to identify potential redox signaling pathways and biological thresholds, which can inform future translational and clinical research. In vitro models are universally employed in biomedical research precisely because they allow isolation of variables that are impossible to dissect in complex living systems. Acknowledgment of Limitations: We fully recognize the inherent differences between in vitro and in vivo environments, including the role of systemic antioxidant capacity, protein binding, and hemodilution—points that are addressed in the Discussion Section. However, these differences do not invalidate the in vitro model; they contextualize its findings. All experimental models involve simplification, and the value lies in how rigorously the system is characterized and how the results are interpreted. Consistency with the Precedent Literature: The methodological approach of diluting pre-prepared O3SS into cell culture media is well established in the field of ozone therapy research and has been used in numerous peer-reviewed studies exploring ozone’s biological effects. Our protocol aligns with these precedents and adds rigorous quantification and dose–response characterization. In addition, the proposed kinetic modeling and mass balance analysis refer to in vitro biochemical analysis, therefore suffering from the same bias indicated and criticized by Franzini et al. In summary, while we appreciate the theoretical concerns raised, the experimental design was grounded in direct measurement, controlled preparation, and a clear mechanistic objective. We believe this provides a robust and valuable contribution to understanding the redox biology of ozone in a cellular context.

As a corollary to the above, a further misunderstanding of the methods used emerges when Franzini et al. state that “the total ozone dose per cell in vitro is drastically higher than that which tissues or plasma components would encounter in vivo, where antioxidant systems, proteins, and lipids immediately quench reactive species”. It is worth noting that the culture medium used contains fetal bovine serum, which provides proteins, lipids, and antioxidants similar to those found in vivo. Thus, the medium itself replicates several quenching factors present in biological fluids. This aspect may not have been considered in the critics’ dose extrapolation.

## 3. Choice of Cellular Models

Cellular in vitro models represent the most widespread method for basic research and phase 1 studies and are also universally accepted as the best strategy for defining cellular pathways under rigorously controlled and repeatable conditions. Franzini et al. dispute the effectiveness and translational validity of studies using established cell lines and primary cultures such as HUVEC. We wish to outline that 6305 papers published in indexed journals have dealt with the BV2 cellular model, and that 624 of those papers were published in *Nature* and related journals, 63 in *Science*, 16 in *The Lancet*, etc. [4,5,6,7,8,9,10,11,12], confirming the suitability of BV2 cells as a model of microglial cells. The two references cited by Franzini et al. to question the BV2 model are relatively older. Since their publication, a large body of literature (including over 6000 papers on BV2 cells, many in high-impact journals) has validated the utility of this cell line as a reproducible model for microglial studies. Moreover, we point out that even primary cultures of microglia differ from the microglial cells present in the central nervous system, as they are isolated from the microenvironment in which they live. These limitations are overcome by adding a series of growth factors that allow for very limited survival in culture, and therefore, these conditions also differ from the in vivo situation but do represent a study model. The Comment appears to criticize primary HUVEC cultures for lacking in vivo complexity, while simultaneously suggesting primary microglial cultures might be more appropriate. In both cases, primary cultures also diverge from in vivo conditions, but they remain useful models when interpreted with appropriate caution.

Furthermore, Franzini et al. say that HUVEC cells (25,094 publications reviewed in PubMed using this cellular model) [13,14] are not a suitable model since they do not replicate the dynamic flow and immune interactions of living tissue. We agree that the in vitro systems do not represent the complexity of the in vivo conditions, but they instead represent a universally accepted model to study certain processes under rigorously controlled, repeatable conditions, with the possibility of analyzing the different variables separately. Refusing experimental models means that new experimental strategies should be tested directly on the patients, a practice that is not acceptable from an ethical point of view.

## 4. Ozone Quantification and Controls

While we acknowledge the importance of precise chemical quantification in ozone studies, we would like to clarify several key aspects of our experimental approach. First, the ozone concentration values used in this study are not based on theoretical assumptions but derive from a previously established and validated method published by our research group. In that earlier work, we employed the same medical ozone generator (WeZONO) and saturation setup under identical experimental conditions to directly measure dissolved ozone concentrations in saline using UV spectrophotometry. The consistent 10% transfer efficiency was empirically confirmed and reported. To clarify, the ozone concentration values were not assumed theoretically but derived from a previously published validation using the same generator and conditions. Referencing those measurements is methodologically appropriate. Second, the nominal concentrations (1, 5, and 10 µg/mL) refer to the generator output, as is standard in the field, while the actual concentrations in the cell medium were explicitly stated after applying the empirically derived 10% transfer factor and the 1:20 dilution. This provides a transparent and reproducible basis for dose–response analysis. We agree that ozone’s reactivity in solution is high, which is precisely why we followed a standardized and previously characterized protocol to ensure consistency. While real-time monitoring during incubation would offer additional detail, the use of a rigorously calibrated system and immediate application after preparation minimizes variability and supports the reliability of the exposure conditions. Finally, the study was designed as a mechanistic investigation into cellular responses under defined ozone-derived oxidative conditions. The concentrations selected span a clinically relevant range reported in ozone therapy research and allow observation of threshold effects and redox signaling behaviors.

The concern that our colorimetric assay for hypochlorite lacked independent calibration and sensitivity validation, and that the conclusion of “negligible” formation was based on the detection limit rather than absolute absence, does not account for the fact that the assay was used to exclude hypochlorite at concentrations below biologically relevant thresholds, which is standard analytical practice and sufficient to rule out confounding effects in our experimental system. First, the Hypochlorite Detection Kit (ab219929, Abcam, Cambridge, United Kingdom) is a commercially validated and widely cited assay, with a specified detection range of 0.1–10 µM hypochlorite and a well-documented sensitivity limit (<0.1 µM) as per the manufacturer’s technical documentation. The assay’s selectivity for ClO^−^ over other reactive oxygen species has been independently verified in peer-reviewed studies. Our adherence to the standardized protocol ensures reproducibility and reliability.

Second, the measured hypochlorite concentration in all ozonated samples was 0.001% of the total oxidative capacity—a value indistinguishable from the baseline level in unconditioned culture medium and at the lower detection limit of the assay. This result was consistent across all ozone concentrations tested, indicating no concentration-dependent formation of hypochlorite during ozonation. From a biological standpoint, even if trace amounts at this detection threshold were present, the corresponding concentration (~0.1 µM) is physiologically negligible and orders of magnitude below the cytotoxic or redox-modulatory range reported in the literature (typically >10 µM). Therefore, it is scientifically unfounded to attribute the observed cellular effects to hypochlorite contamination. While additional calibration data could be presented in a supplementary form, the use of a validated commercial kit, internal controls, and replication in accordance with standard laboratory practice provides sufficient evidence to confidently exclude hypochlorite as a significant confounder in this study. The assertion that hypochlorite formation was negligible is therefore both analytically and biologically justified. We stand by the methodological rigor and validity of this control experiment.

## 5. Gene Expression and Biological Interpretation

We thank the authors for recalling the dynamic plasticity of microglial cell polarization, which occurs through multiple steps as described in numerous papers using in vitro systems. The analyzed pathways, confirmed in many of our previous papers, summarize a process that, although characterized by different steps, leads towards a pro-inflammatory or anti-inflammatory phenotype, depending on the microenvironmental conditions. This is evidently a dichotomy, as noted by Franzini, Chirumbolo et al. [2], which represents two opposite functional states, also characterized by a different morphology. The aim of this article is evidently not to delve into the different steps of the process but to demonstrate the anti-inflammatory effect of ozonized solutions. Therefore, some fundamental characteristics of the pro- and anti-inflammatory phenotypes have been examined to confirm this effect, such as the induction of cytokine gene expression and some markers of the different polarization states. Other functional characteristics, which we have examined in previous work, were not central to the present study’s focused objective and were therefore omitted for brevity. In this regard, several references on the subject, both ours and those of other international research groups, can be cited [15,16].

## 6. Statistical Analysis

We appreciate the opportunity to clarify the statistical approach used in our study, which we believe adheres to widely accepted standards in experimental cell biology and molecular pharmacology. First, regarding sample size and reported *p*-values: the experiments were conducted with at least three independent biological replicates, each comprising multiple technical replicates. This design is standard in the field for in vitro mechanistic studies and provides sufficient power to detect biologically relevant effects when effect sizes are large and variability is controlled, as was the case in our experiments. Regarding the statistical concerns raised, low *p*-values in our study arise from low intra-group variance and large effect sizes, which are typical in well-controlled qPCR and ELISA experiments. Multiple comparison corrections were not required because the comparisons were planned and hypothesis-driven, not exploratory. This approach follows standard practice in experimental cell biology. When multiple comparisons were relevant within a given figure, significance levels were clearly indicated (*, #) and interpreted conservatively. Third, expression of mRNA via qPCR is a highly sensitive and quantitatively precise method, known for its low variability when normalized to appropriate housekeeping genes and performed with rigorous technical controls. The large fold-changes observed, combined with minimal standard deviations, naturally yield high statistical significance even with (*n* = 3) biological replicates—a fact well documented in the molecular biology literature. In summary, the statistical analysis was chosen to match the experimental design and biological questions. The results are presented transparently, with measures of variance clearly reported, and the conclusions are supported by consistent evidence across complementary assays. We are confident that the statistical approach is sound and that the reported significance values truthfully reflect the robustness of the observed effects.

## 7. Comparative Agents

Regarding the request for additional pharmacological controls, such as dexamethasone or N-acetylcysteine (NAC): while such compounds are indeed valuable tools in mechanistic research, their inclusion was beyond the scope of this particular study. Our primary objective was to characterize the direct cellular response to ozone in solution and its interaction with an LPS-induced inflammatory model—not to perform a comparative pharmacological benchmarking against established anti-inflammatory or antioxidant agents. The absence of such controls does not invalidate the observed effects of ozone; rather, it delineates the focused nature of the experimental design. Furthermore, the saline-only control serves precisely to distinguish nonspecific or vehicle-related effects from those attributable to ozone. Given that hypochlorite—a potential reactive byproduct—was ruled out as a significant contributor, and considering the immediate reactivity of ozone in saline, it is methodologically sound to attribute the observed biological changes primarily to ozone-derived reactive oxygen species and their subsequent signaling effects. Naturally, scientific knowledge is cumulative, and experimental designs can be expanded infinitely in complexity. However, the value of a controlled, hypothesis-driven study lies in its ability to clarify specific mechanisms—not to exhaust all possible comparisons. Our work successfully elucidated part of the mechanism by which ozone in saline acts in cellular models, providing a necessary foundation for future research that may include the comparative agents you mention. We maintain that the experimental approach, with its defined controls and focused objectives, is valid and contributes meaningfully to the understanding of ozone’s biological activity in vitro.

## 8. Mechanistic Relevance and Translational Context

We would like to address your remarks with a clear, evidence-based perspective on the current scientific landscape regarding ozone therapy. First, it is widely accepted that ozone—like many medical gases and therapeutic agents—exhibits dose-dependent and route-dependent effects. At inappropriate concentrations or via inhalation, it can indeed be toxic. However, the therapeutic application of ozone in medicine, specifically via controlled modalities, operates within precisely defined, low-dose ranges that have been extensively studied for safety and biological activity. Your assertion that supportive references derive predominantly from “low-impact journals or self-cited works” is inaccurate and dismissive of a substantial and growing body of the peer-reviewed literature. To illustrate, a search in authoritative databases reveals:

- A total of 23,471 studies indexed in PMC (PubMed Central, NIH, USA).
- A total of 5166 references in MEDLINE (PubMed, NIH, USA).
- Among these, 278 randomized controlled trials, 115 systematic reviews, and 63 meta-analyses specifically address medical ozone applications.

Furthermore, 126 clinical trials are registered on ClinicalTrials.gov, investigating ozone across various pathologies—a clear indicator of its recognition as a legitimate subject of clinical research. The phrase “safe and well tolerated” in our manuscript is not anecdotal; it is supported by this cumulative clinical evidence, particularly in the context of systemic ozone administration via major autohemotherapy or O3SS, where adverse events are rare and mild when protocols are followed. Regarding mechanistic plausibility, the concept of “oxidative eustress” is well established in redox biology, referring to the ability of mild, controlled oxidative stimuli to activate adaptive cellular responses without causing damage. This is not promotional language but a scientific framework supported by studies on other medical gases (e.g., hydrogen sulfide and nitric oxide) and preconditioning phenomena. While we agree that further pharmacokinetic and mechanistic studies are valuable—and in fact represent the very rationale for our in vitro investigation—the existing evidence sufficiently justifies the exploration of ozone as a potential adjunctive therapy. Our study contributes precisely to this mechanistic understanding by examining redox signaling in a controlled cellular model. We therefore stand by the balanced and evidence-informed presentation of ozone therapy in our manuscript, which aligns with the current scientific literature and encourages further rigorous research.

## 9. Limitations and Balanced Interpretation

The dose–response experiments are reported in Figure 1 of the original manuscript [1], in which a possible cytotoxic effect induced by the addition of ozonized solutions with different ozone concentrations is analyzed. Franzini et al. are certainly correct in stating that the response to ozone treatment is acute, as is also the case in clinical settings, where, according to them, multiple therapeutic interventions are required over time. Ozone treatments are used to re-establish conditions of homeostatic equilibrium, conditions that are disrupted during various pathologies and can be restored by treatments that address oxidative stress and inflammatory processes. The aim of this work was to demonstrate that ozonized solutions can target these processes and promote a shift toward equilibrium. We also underline that transcriptional changes represent beneficial modulation depending on the ozone-induced acute stress responses, as also occurs in vivo. Moreover, since Franzini et al. argued that results obtained in vitro cannot be extrapolated to the clinic, why do they ask to perform some replication of exposure to mimic repeated therapeutic sessions?

The critique argues, of Franzini et al. [2], that extrapolating in vitro results to systemic therapeutic mechanisms is unjustified, because ozone chemistry in microwell cell cultures is fundamentally different from that occurring during in vivo O3SS infusion, where ozone reacts immediately with plasma components to form short-lived secondary species; the absence of discussion on ozone decay kinetics, solubility limits, and heterogeneous reactivity is therefore considered a major conceptual flaw that undermines the study’s premise. However, the argument presented appears to overlook the well-established pharmacological paradigm of ozone’s mechanism of action in biological systems—a paradigm that is fundamental to interpreting both in vitro and in vivo findings. Ozone is widely recognized in the scientific literature not as a direct pharmacologically active molecule, but as a “pro-drug” or “pro-oxidant” that acts indirectly. Its biological effects are mediated through its immediate reaction with biological substrates present in physiological fluids and cell membranes—including unsaturated lipids, antioxidants, and proteins—generating a cascade of reactive oxygen species (ROS) and lipid oxidation products (LOPs), such as hydrogen peroxide (H_2_O_2_) and 4-hydroxy-alkenals. These secondary messengers, not ozone itself, are responsible for activating specific redox-sensitive signaling pathways (e.g., Nrf2, NF-κB, MAPK) and modulating transcriptional activity. This indirect mechanism has been consistently documented in peer-reviewed studies, and it forms the conceptual backbone of our experimental design.

Consequently, the in vitro model used in our study—where ozone reacts directly with cell culture medium and cellular components—faithfully reproduces this initial oxidative event and the generation of the very same signaling mediators (H_2_O_2_, LOPs) that are postulated to act systemically in vivo. The fact that ozone itself decomposes rapidly is not a flaw in the model, but rather a reflection of its intended mechanism: to trigger a controlled oxidative challenge whose downstream messengers can be studied in isolation.

Therefore, the assertion that our in vitro findings are irrelevant to systemic outcomes is scientifically unfounded. On the contrary, our experimental setup isolates the primary redox-signaling step that is essential and common to both local and systemic ozone effects. We encourage a review of the extensive literature on ozone’s indirect mechanism before dismissing the translational relevance of controlled cellular models. These media, in fact, contain amino acids, vitamins, glucose, and are added with bovine serum, which provides the factors listed by the authors.

## 10. Conclusions

We respectfully disagree with the characterization of our discussion as a ‘justification of an already held belief.’ Below, we outline how the discussion systematically addresses confounding factors, acknowledges limitations, and anchors conclusions in data rather than advocacy. The discussion is a rigorous and balanced interpretation of the experimental data within the established scientific framework of ozone therapy and redox biology. A careful reading of the discussion reveals several critical elements that contradict this claim:

1. Detailed mechanistic contextualization. We systematically address potential confounding factors, including:
  - The absence of cytotoxic byproducts (hypochlorite and chlorates) based on prior analytical studies [17,18] and our own experimental validation.
  - The explicit ruling out of hydroxyl radical formation via Fenton chemistry due to the lack of detectable H_2_O_2_ and the stringent limits of iron content in pharmacopeia-grade saline.
  - The acknowledgment of the indirect, pro-drug mechanism of ozone, where its biological effects are mediated by secondary messengers (ROS and LOPs) acting on specific signaling pathways (Nrf2 and PPARγ).
2. Explicit acknowledgment of limitations and modality comparisons. Far from minimizing constraints, we provide a dedicated and nuanced comparison of O3SS with other administration routes. We explicitly state:
  - The significant disadvantage of O3SS regarding lack of standardization.
  - The recognition that probably major autohemotherapy (MAH) remains the preferred method for systemic ozone therapy due to its superior efficacy, safety, and reproducibility.
  - This balanced comparison (advantages vs. disadvantages of O3SS vs. MAH vs. glucose infusion) demonstrates a critical, not promotional, evaluation of the clinical applicability of our in vitro findings.
3. Data-driven, not belief-driven, conclusions. Our interpretation is directly anchored to the observed results:
  - The anti-inflammatory polarization of BV2 microglia (downregulation of IL-1β and iNOS; upregulation of IL-10 and Arg-1) is presented as a data point, followed by a plausible mechanistic link to known pathways (PPARγ activation, Nrf2 signaling).
  - The proliferative effect on HUVECs is cautiously linked to a potential mechanism suggested by independent clinical observation (CD34+ mobilization), clearly framed as a hypothesis for further investigation.
4. Integration within the existing scientific consensus. The discussion places our findings within the well-documented concept of oxidative eustress and redox bioregulation—a recognized paradigm in physiology and pharmacology for other medical gases (e.g., NO, H_2_S). Citing key reviews and preclinical studies (e.g., Bocci, León Fernández et al.) provides a legitimate theoretical scaffold, not an act of advocacy.

In summary, our discussion critically interprets the data by

- Confronting potential artifacts.
- Placing results in the context of established mechanisms.
- Honestly discussing the limitations and comparative standing of the tested modality.
- (O3SS)—proposing mechanistic hypotheses for the observed effects.

Therefore, the assertion that the discussion is non-critical or promotional is not supported by the actual content of the manuscript. We have provided a measured, evidence-based analysis that accurately reflects the scope and implications of our in vitro study.
